# Management of rifampicin-resistant tuberculosis in conflict-affected areas: The case of Iraq

**DOI:** 10.1371/journal.pone.0296952

**Published:** 2024-01-19

**Authors:** Hiwot Melak Tesfahun, Layth Al-Salihi, Nadia Abdulkareem Al-Ani, Ahmed Asmer Mankhi, Ammar Mohammed, Chenery Ann E. Lim, Riadh Abdulameer Al-Hilfi, Christelle G. Jouego, Tom Decroo, Krystel Moussally, Gabriella Ferlazzo, Petros Isaakidis

**Affiliations:** 1 Médecins Sans Frontières (MSF), Operational Center Brussels, Brussels, Iraq; 2 National TB Institute, Ministry of Health, Baghdad, Iraq; 3 MSF, Operational Center Brussels, Iraq Project, Baghdad, Iraq; 4 Iraq Public Health Directorate, Baghdad, Iraq; 5 Molecular Diagnostic and Research Group, University of Yaoundé I, Soa, Cameroon; 6 Institute of Tropical Medicine, Department of Clinical Sciences, Unit of HIV and Tuberculosis, Antwerp, Belgium; 7 MSF, Lebanon Branch Office, Middle East Medical Unit, Beirut, Lebanon; 8 Southern African Medical Unit, Médecins Sans Frontiers, Cape Town, South Africa; 9 Clinical and Molecular Epidemiology Unit, Department of Hygiene and Epidemiology, University of loannina School of Medicine, loannina, Greece; Kings College Hospital, UNITED KINGDOM

## Abstract

Since December 2019, the World Health Organization (WHO) has encouraged National Tuberculosis Programs to deprioritize the use of injectable-containing regimens and roll-out all-oral bedaquiline-containing regimens for rifampicin-resistant tuberculosis (RR-TB) treatment. Consequently, Iraq gradually replaced the injectable-containing regimen with an all-oral regimen, including bedaquiline. To assess treatment enrolment and outcomes of both regimens during a transitioning phase in Iraq, where health system services are recovering from decades of war, we conducted a nationwide retrospective cohort study using routinely collected programmatic data for patients enrolled between 2019–2021. We describe treatment enrolment and use logistic regression to identify predictors of unfavorable treatment outcomes (failure, death, or lost to follow-up), including regimen type. Nationwide, a total of 301 RR-TB patients started treatment, of whom 167 concluded treatment. The proportion of patients enrolled on the all-oral regimen increased from 53.2% (50/94) in 2020, to 75.5% (80/106) in 2021. Successful treatment was achieved in 82.1% (32/39) and 63.3% (81/128), for all-oral and injectable-containing regimens respectively. Moreover, the proportion of lost to follow-up was lower among those treated with the all-oral versus the long injectable-containing regimen; respectively 2.6% (1/39) versus 17.9% (23/128: p = 0.02). Unfavorable treatment outcome was associated with male gender (aOR 2.12, 95%CI:1.02–4.43) and age <15 years (vs 30–49 years, aOR 5.80, 95%CI:1.30–25.86). Regimen type (aOR 2.37, 95%CI: 0.91–6.13) was not significantly associated with having an unfavorable treatment outcome. In Iraq, the use of bedaquiline-containing all-oral regimen resulted in a high treatment success and reduced lost to follow-up.

## Introduction

Worldwide an estimated 10.6 million people fell ill with tuberculosis (TB) in 2021, including 450 000 with rifampicin-resistant tuberculosis (RR-TB) [1]. Among patients diagnosed with RR-TB, 36% started effective second-line TB treatment, with a success rate of 60% [1]. Management of RR-TB patients has been evolving over the years. For decades, injectable-containing regimens (i.e., capreomycin (Cm), kanamycin (Km), and amikacin (Am)) were the mainstay of treatment for RR-TB. The use of Cm and Km is associated with irreversible hearing loss and worse treatment outcome when used for longer duration [2–6]. Since 2019, the World Health Organization (WHO) recommends countries to gradually scale-up all-oral regimens containing bedaquiline (BDQ) in combination with an effective background regimen [7–10]. This recommendation is informed by evidence on the safety and effectiveness of all-oral BDQ-containing regimens, also in programmatic settings [11–17].

Iraq is considered a moderately burdened country for RR-TB. An estimated 4.8% of new and 38.0% of previously treated people with pulmonary TB were estimated to have RR-TB in 2021. Moreover, 110 people with RR-TB were notified by the TB program to WHO during the same year [1]. Until 2020, RR-TB treatment was based on a 18-24-month injectable-containing regimen using a centralized model of care, managed by the National TB institute (NTI). In 2020, in collaboration with Médecins Sans Frontières (MSF), the first RR-TB guideline was developed. It incorporated WHO recommendations on all-oral regimens [9, 18]. In the same year, MSF supported the NTI in rolling out a 18-20-month all-oral BDQ-containing regimen [7]. Additionally, MSF also supported the implementation of patient support packages, including education and counselling, provision of food packages, transport reimbursement, and adverse event (AE) monitoring and management.

Iraq is recovering from decades of protracted war and conflict, with a fragile health care system still struggling to provide care for communicable diseases such as RR-TB. In addition, there is limited availability of trained health care professionals and adequate infrastructure dedicated to TB control services. Our study therefore aimed to describe how RR-TB care was provided in Iraq. We described RR-TB treatment enrolment, treatment outcomes, and estimate predictors for unfavorable treatment outcome.

## Materials and methods

### Design and study population

This is a retrospective cohort study of all RR-TB patients started on any of the available treatment regimens in Iraq, between 1^st^ January 2019 and 31^st^ December 2021. Data was collected until 31 May 2022 and accessed for research purposes in June 2022.

### Study setting

Iraq is part of the WHO Eastern Mediterranean Region (EMR) with an estimated population of 40 million, spread over 19 governorates or regions [18, 19]. The country has seen many wars over the past decades. Successively, the country witnessed destruction of health care services, fleeing of health care workers, economic stagnation, and reduced access to essential services [20]. Based on a humanitarian needs review published by the United Nations Office for the Coordination of Humanitarian Affairs in March 2022, 2.5 million people in Iraq needed some form of humanitarian assistance, including food, water and sanitation, housing and access to health care among others [21]. Despite a recovering health system, Iraq continues to suffer from the long-term effects of the war with a high burden of communicable diseases, such as TB. The NTI, established in 1989, adopted the WHO End TB strategy to reduce TB burden, TB related morbidity and mortality by ensuring universal access to TB diagnosis, care and treatment, including care for RR-TB [18].

MSF started collaborating and providing technical support to the NTI in 2018, through capacity building, clinical consultation, strengthening the National Reference Laboratory (NRL) and provision of RR-TB medicines and laboratory reagents. Since 2020, MSF also supported the introduction of regular patient support services. In addition, MSF provided a trained counsellor to fill the human resources gap at the NTI.

### Diagnosis and treatment of RR-TB

Thirty health facilities provide RR-TB diagnosis across the country. More precisely, 19 Consultant Chest and Respiratory Disease clinics (CRDC) in each governorate, the NRL, and ten of the 131 TB coordinator units. Presumed RR-TB patients, such as contacts of known RR-TB and previously treated patients, are referred by the TB coordinator units to one of the CRDCs to have their sputum tested using the Xpert MTB/RIF® (Cepheid, Sunnyvale, CA, USA; Xpert MTB/RIF) platform. Patients with confirmed RR-TB are referred to the single RR-TB treatment centre of NTI, located in Bagdad, for RR-TB treatment initiation and monitoring. The NRL, located in the same area as the NTI, provides services, such as first- and second-line probe assay (LPA) testing, and culture followed by drug susceptibility testing (DST) at baseline and during follow-up. Other baseline and monitoring investigations provided to RR-TB patients are listed in Table 1. Clinical diagnosis of RR-TB patients is made by the clinician when bacteriological confirmation is not possible (e.g., for children) among patients with signs of presumptive RR-TB, taking into consideration contact with RR-TB patients and/or lack of response to repeated first-line TB treatment.

**Table 1:** Baseline and follow-up schedule for clinical and bacteriologic investigations of RR-TB patients enrolled on either all-oral or injectable-containing regimen between 2019–2021, Iraq.

**Baseline examination (month 0) and monthly follow-ups (until 20–24 months)**	Clinical evaluation, weight, complete blood count, AST/ALT, serum creatinine, serum electrolytes, smear examination, culture, Ishihara test, RBS (only for patients with diabetes), pregnancy test (for female patients of childbearing age)
**At treatment initiation only**	HIV test
**At 2 weeks, month 1, then monthly during the intensive phase with BDQ**	ECG
**At 6 month and end of treatment**	CXR
**At time of presumed or declared treatment failure**	SL-DST; 2^nd^ LPA

AST/ALT = aspartate amino transferase/ alanine amino transferase, CXR = chest X-ray, ECG = electrocardiography, HIV = Human Immunodeficiency virus, 2^nd^ LPA = second-line probe assay, M = monthly, M1 = month 1, RBS = random blood sugar, SL-DST = second-line drug-susceptibility test.

From 2010 till 2020, the NTI was the only treatment centre for RR-TB nationwide. RR-TB patients diagnosed from various parts of the country had to travel to the NTI for treatment initiation and for baseline and follow-up tests following national guidelines [18]. One RR-TB expert physician is responsible for ambulatory patient management. In 2021, the NTI took the first step towards RR-TB treatment initiation decentralization to the CRDC outside Baghdad. To date no centre provides the full RR-TB care package outside Baghdad.

### Clinical management and follow-up

RR-TB care is provided free of charge in Iraq. Baseline pre-therapeutic and follow-up clinical and bacteriologic investigations were performed at the NTI (See Table 1). RR-TB patients visited the clinic on a (bi-) monthly basis for drug refills as well as treatment monitoring.

Initially, adverse event monitoring was limited. Audiometry was not available in the centre. Patient support activities were non-existent, except for occasional food packages which were provided to patients by the International Organization for Migration. As from January 2020 onwards, AE screening and management was conducted, however, data was not systematically collected. The patient support package provided included pre-therapeutic education and counselling, follow-up of patients who interrupted treatment via phones, screening for nutritional needs, provision of a food basket and transport reimbursement. These patient support packages focused on patients starting the all-oral regimen which later expands to include all RR-TB patients regardless on the treatment regimen. After treatment initiation, patients on the all-oral BDQ-containing regimen would then self-administer oral drugs at home, with or without family support, while patients who received the injectable-containing regimen had to go to the nearest health facility to get their daily injections. Incarcerated individuals with RR-TB received their injections at the prison health centre and data reporting was done by the prison healthcare worker.

### Treatment regimens

From 2010 to 2019, the standard 18–24 months injectable-containing regimen was the only available regimen to treat all RR-TB patients in Iraq, for adults and children. Moreover, in case of poor response, the regimen was prolonged for another four months.

Starting January 2020, with support from MSF, Iraq introduced the WHO recommended, all-oral regimen lasting 18–20 months. Initially, this regimen was offered to RR-TB patients not responding clinically and bacteriologically to the injectable-containing regimen. Patients either had resistance to fluroquinolone (FQr) or second-line injectable (SLI) (SLIr)), or showed intolerance to the injectables, or were children or pregnant women. Since 2021, the majority of RR-TB patients were enrolled on the all-oral regimen. The injectable-containing regimen was still provided to patients who had no access to baseline and follow-up monitoring, such as patients who were treated for RR-TB while being in prison. Details of regimen composition are summarized in Table 2.

**Table 2:** Composition, indications, and duration of both the all-oral and injectable-containing regimens in Iraq between 2019–2021.

Indications	Composition of the regimen
**All-oral 18–20 months regimen: Since 2020**	
**Majority of adults with RR-TB**	6 months of BDQ, LFX, LZD, CFZ
	Plus 12–14 months of LFX, LZD, CFZ, Cs*
**Children**	6 months of DLM, LFX, LZD, CFZ
	Plus 12 months: LFX, LZD, CFZ
**FQ/SLI resistant**	18–20 months BDQ, DLM**, LZD, CFZ
**18–24 months injectable-containing regimen: 2019–2021**	
**All RR-TB patients**	6–8 months Cm /Am, LFX, Eto, Cs, Z, (E)
	Plus 14–16 months: LFX, ETO, CS, Z, (E)

Am = amikacin, BDQ = bedaquiline, CFZ = clofazimine, Cm = capreomycin, DLM = delamanid, Eto = ethionamide, FQr = fluroquinolone resistant, LFX = levofloxacin, LZD = linezolid, MDR-TB = multi-drug-resistant tuberculosis, RR = rifampicin-resistant, SLI = second-line injectable resistant, TB = Tuberculosis, Z = pyrazinamide

*Cycloserine (Cs) was added at the beginning for some patients as a 5^th^ drug or substituted by either LZD or CFZ if intolerance to these drugs developed.

** DLM could be stopped the earliest at 6 months to continue with BDQ, CFZ and LZD until the end of treatment. The clinician decided on the duration based on bacteriological and clinical response on a case-by-case basis.

### Data collection and variables

De-identified data was compiled and extracted from the routinely updated NTI and MSF Excel databases (Excel, version 2206). Data that was incomplete in the electronic file was retrieved from the medical files. Demographic and clinical characteristics collected includes age, sex, type of baseline resistance, and comorbidities (HIV and diabetes). Data on AE monitoring and management was not systematically collected and recorded in the electronic database, and therefore not reported in this study. Treatment outcome variables were collected and followed WHO and national RR-TB definitions [18, 22, 23]. Favorable outcomes include cure and treatment completion and unfavorable outcomes were lost to follow-up (LTFU) during treatment, death and treatment failure. Failure was defined as when: treatment is terminated or there is a need for a permanent regimen change of at least two anti-TB drugs because of lack of culture conversion by the end of the intensive phase, bacteriological reversion in the continuation phase after conversion to negative, evidence of additional acquired resistance to FQs or SLI drugs, or adverse drug reactions [24]. However, regimen change due to AEs were not systematically recorded in the NTI database. We reported patients having diabetes mellitus, those either self-reported having diabetes or who received a positive test based on the clinician’s decision.

### Statistical analysis

Baseline demographics and clinical characteristics were analysed using summary statistics (medians with interquartile range (IQR) for continuous variables, frequencies, and proportions for categorical ones). Outcome analyses were performed on patients who had a recorded outcome at the end of the study follow-up period (31 May 2022). Predictors of unfavorable outcome were evaluated using bivariable and multivariable regression models. A saturated multivariable model was stepwise simplified and used until the multivariable model only included those variables that were significantly associated (P-value ≤ 0.05) with treatment outcome. The variable of interest (type of regimen) was included in the model, regardless of the level of association. Results were reported using odds ratio (OR) and adjusted odds ratios (aOR) with their 95% confidence interval (95% CI). The data was analysed using R-studio version 4.2.0 (2022-04-22).

### Ethical approval

The research protocol was approved by the Institutional Review Board (IRB) of the Institute of Tropical Medicine (reference number: 1571/22, March 28, 2022) and the Public Health Directorate/Training and Research, Iraq (reference number: 1015, 31 May 2022). The study fulfilled the exemption criteria set by MSF ethics review board (Geneva, Switzerland) for a posterior analysis of routinely collected clinical data and thus did not require MSF ERB review (reference number 2229, April 6, 2022). Due to the retrospective nature of the study, we received a waiver of the individual informed consent from the ERB. The benefits for future RR-TB patients, for whom care will be informed by lessons learnt from the present study, outweigh the risks, which are considering minimal given that data is pseudonymized.

## Results

**Fig 1:**
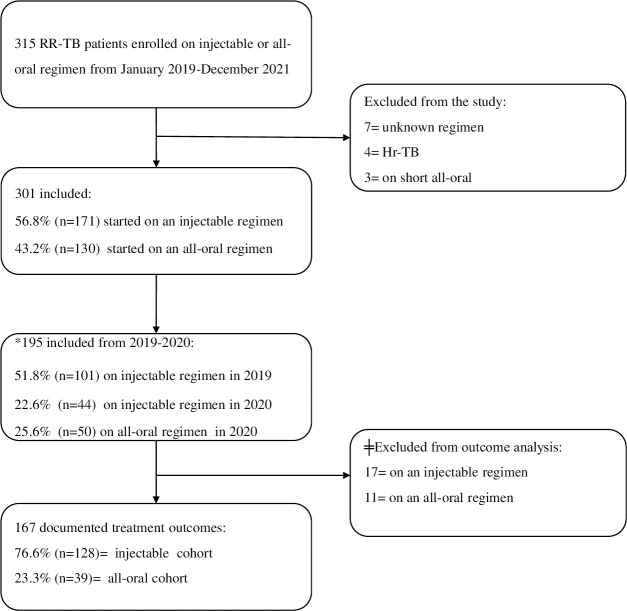
Enrolment flow chart of RR-TB patients. Hr-TB = isoniazid resistant tuberculosis, RR-TB = rifampicin-resistant tuberculosis, ǂ 28/195 (14.3%) patients were still on treatment as of 31 May 2022. *Patients for 2021 cohort were not included in the outcome analysis.

From 1^st^ January 2019 to 31^st^ December 2021, a total of 315 patients were enrolled on a RR-TB treatment regimen. Of this, 4.4% (n = 14) patients were excluded from the study, either because they had isoniazid-resistant TB without resistance to rifampicin (Hr-TB), received a short all-oral regimen (4–6 BDQ-LFX-Eto-E-Z-high dose isoniazid (hH)-CFZ/5 LFX-CFZ-Z-E) or the regimen type was not documented. Of the 301 patients included in the study, 56.8% (n = 171) started the injectable-containing regimen and 43.2% (n = 130) the all-oral regimen (Fig 1).

### Baseline characteristics

The median age was 36 years old IQR [25–47] with the majority being between 30 and 49 years old (n = 135, 44.8%). Patients newly diagnosed with TB represented 39.2% (118/301). None of the patients were HIV positive while 12.6% (38/301) had diabetes mellitus. Most patients (56.8%; 171/301) started the injectable-containing regimen while 43.2% (130/301) were put on the all-oral regimen. From 2020 to 2021, there has been progressive roll-out of the all-oral regimen from 53.2% (50/94) in 2020, to 75.5% (80/106) in 2021. Among patients who received the all-oral regimen, 19.2% (25/130) were previously treated with second-line TB drugs, compared to 0.6% (1/171) among patients receiving the injectable-containing regimen. All incarcerated individuals with RR-TB, 12.3% (37/301) received the injectable-containing regimen and 9.3% (28/301) started treatment without bacteriological confirmation of RR-TB. FQr was diagnosed in 6.6% (20/301) of patients while 77.4% (233/301) had missing SL DST results (Table 3).

**Table 3:** Baseline characteristics of RR-TB patients enrolled on all-oral and injectable-containing regimen between 2019–2021 in Iraq.

Variables	TOTAL	Injectable regimen	All-oral regimen
	(N = 301)	(N = 171)	(N = 130)
Gender n (%)			
Male	185 (61.5)	116 (67.8)	69 (53.1)
Female	116 (38.5)	55 (32.2)	61 (46.9)
**Age -years (median, IQR)**	36 [25–47]	36 [22–47]	39 [26–48]
**Age group (years) n (%)**			
<15	17 (5.6)	5 (2.9)	12 (9.2)
15–29	79 (26.2)	47 (27.5)	32 (24.6)
30–49	135 (44.8)	80 (46.8)	55 (42.3)
≥50	70 (23.3)	39 (22.8)	31 (23.9)
**Type of TB n (%)**			
Pulmonary	266 (88.4)	156 (91.2)	110 (84.6)
Extra-pulmonary	35 (11.6)	15 (8.8)	20 (15.4)
Previous treatment n (%)			
New case	118 (39.2)	83 (48.5)	35 (26.9)
Previously treated			
*With FLD*	148 (49.2)	78 (45.6)	70 (53.8)
*With SLD*	26 (8.6)	1 (0.6)	25 (19.2)
No data	9 (3.0)	9 (5.3)	0 (0.0)
HIV status n (%)			
Positive	0 (0.0)	0 (0.0)	0 (0.0)
Negative	211 (70.1)	81 (47.4)	130 (100.0)
No data	90 (29.9)	90 (52.6)	0 (0.0)
Diabetes n (%)			
Yes	38 (12.6)	14 (8.2)	24 (18.5)
No	263 (87.4)	157 (91.8)	106 (81.5)
Incarcerated n (%)			
Yes	37 (12.3)	37 (21.6)	0 (0.0)
No	264 (87.7)	134 (78.4)	130 (100.0)
Pregnancy reported n (%)	4 (1.3)	0 (0.0)	4 (1.3)
RR-TB diagnosis n (%)			
Bacteriologically confirmed	273 (90.7)	156 (91.2)	117 (90.0)
Clinically diagnosed	28 (9.3)	15 (8.8)	13 (10.0)
FQ DST n (%)			
FQr	20 (6.6)	1 (0.6)	19 (14.6)
FQ-sensitive	48 (15.9)	5 (2.9)	43 (33.1)
FQr unknown	233 (77.4)	165 (96.5)	68 (52.3)
Baseline culture n (%)			
Positive	188 (62.4)	105 (61.4)	83 (63.8)
Negative	30 (10.0)	12 (7.0)	18 (13.8)
No data	83 (27.6)	54 (31.6)	29 (22.3)
Year of enrolment n (%)			
2019	101 (33.5)	101 (100.0)	0 (0.0)
2020	94 (31.2)	44 (46.8)	50 (53.2)
2021	106 (35.2)	26 (24.5)	80 (75.5)

FQ = fluoroquinolone, FQr = fluoroquinolone resistant, FLD = First-line drugs, IQR = interquartile range, MDR = multi-drug resistant, Pre-XDR = pre-extensively-drug resistant, RR = rifampicin-resistant, SLD = second-line drugs, TB = tuberculosis

### Treatment outcomes

Outcome analysis was conducted for a cohort of 195 RR-TB patients enrolled between 2019–2020 on either the injectable-containing or the all-oral regimen. Among those, 85.6% (167/195) had a documented treatment outcome at the end of the study period. Among patients initiated on treatment, 63.3% (81/128) and 82.1% (32/39) had favourable treatment outcome after receiving respectively the injectable-containing and the all-oral regimen. Unfavorable treatment outcome was recorded in 35.9% (46/128) and 17.9% (7/39) of patients who received respectively the injectable-containing and the all-oral regimen. Treatment failure was recorded for 5.1% (2/39) on the all-oral regimen. Both had baseline FQr. Out of the two patients with treatment failure during the all-oral regimen, one continues an all-oral (21BDQ-DLM-CFZ-LZD) regimen as no other options were available while the other one died. See the treatment outcomes per type of regimen in Table 4.

**Table 4:** Treatment outcomes of RR-TB patients who completed either the injectable-containing or the all-oral regimen before 31 May 2022 in Iraq.

Treatment outcome	Outcome available	Injectable regimen	All-oral regimen	*P-value
	N = 167*	N = 128	N = 39	
**Cured**	70	44 (34.4)	26 (66.7)	
**Treatment completed**	43	37 (28.9)	6 (15.4)	
**Failure**	12	10 (7.8)	2 (5.1)	
**LTFU**	24	23 (17.9)	1 (2.6)	
**Died**	17	13 (10.2)	4 (10.2)	
**Not evaluated**	1	1 (0.8)	0 (0.0)	
**Treatment success**	113	81 (63.3)	32 (82.1)	0.03
**Unfavorable outcome**	53	46 (86.8)	7 (13.2)	

LTFU = lost to follow-up, RR-TB = rifampicin-resistant tuberculosis, treatment success: cured and treatment completed; unfavorable outcome: (failure, lost to follow-up, and died)

* Fisher’s exact test or chi-squared test as relevant

Nine children (< 15 years) were included in the outcome analysis. Among them, four (44.4%) received an all-oral regimen and five an (55.6%) injectable regimen. Of nine, six had an unfavorable treatment outcome and death was recorded in 50% (n = 3) of them (two under the all-oral and one under the injectable-containing regimen). Among children who died, two were less than or equal to five years old and one had a previous treatment history with an injectable-containing regimen and disseminated TB (abdomen, pulmonary). The median time to death from treatment initiation was 30 days.

### Predictors of unfavorable treatment outcome

Unfavorable treatment outcome was associated with male sex (aOR 2.12, 95%CI 1.02–4.43), and being younger than 15 years compared to 30 to 49 years old (aOR 5.80, 95%CI 1.30–25.86) (Table 5). The use of the injectable-containing regimen was associated with having an unfavorable outcome in the bivariable analysis (OR 2.47, 95%CI 1.03–5.91), but not in the multivariable analysis (aOR 2.37, 95%CI 0.91–6.13).

**Table 5:** Predictors of unfavorable treatment outcome among RR-TB patients started on the injectable-containing or the all-oral regimen between 2019–2020, Iraq.

	Total	Favorable	Unfavorable	OR	[95%CI]	aOR	[95%CI]	p-value#
	(N = 166) ¥	(N = 113)	(N = 53)					
**Gender**								
Male	94	56 (59.6)	38 (40.4)	2.53**	[1.26,5.06]	2.12*	[1.02,4.43]	<0.01
Female	72	57 (79.2)	15 (20.8)	1		1		
**Age group**								
<15	9	3 (33.3)	6 (66.7)	4.72*	[1.15,19.39]	5.80*	[1.30,25.86]	<0.05
15–29	59	46 (78.0)	13 (22.0)	0.74	[0.33,1.68]	0.95	[0.40,2.26]	NS
30–49	61	44 (72.1)	17 (27.9)	1		1		
> = 50	37	20 (54.1)	17 (45.9)	2.17	[0.93,5.05]	2.18	[0.91,5.21]	NS
**Previous FL treatment**						NS		
No	71	51 (71.8)	20 (28.2)	1				
Yes	95	62 (65.3)	33 (34.7)	1.35	[0.69,2.61]			
**Previous SL-treatment**						NS		
No	154	104 (67.5)	50 (32.5)	1				
Yes	10	7 (70.0)	3 (30.0)	0.97	[0.26,3.59]			
NA	2	2 (100.0)	0 (0.0)	0.41	[0.02,8.78]			
**Diabetes**						NS		
No	146	98 (67.1)	48 (32.9)	1				
Yes	20	15 (75.0)	5 (25.0)	0.72	[0.26,2.02]			
**Incarcerated**								
Yes	6	6(100)	0(0)					
No	160	107(66.8)	53(33.2)	NA		NA		
**Site of TB**						NS		
Pulmonary	151	107 (70.9)	44 (29.1)	1				
Extra-pulmonary	15	6 (40.0)	9 (60.0)	3.53*	[1.23,10.16]			
**Clinically diagnosed**						NS		
No	151	105 (69.5)	46 (30.5)	1				
Yes	15	8 (53.3)	7 (46.7)	2	[0.71,5.67]			
**Enrolment year**							NS	
2019	100	68 (68.0)	32 (32.0)	1				
2020	66	45 (68.2)	21 (31.8)	1	[0.51,1.93]			
**Type of regimen**								
All-oral	39	32 (82.1)	7 (17.9)	1		1		
Injectable	127	81 (63.8)	46 (36.2)	2.47*	[1.03,5.91]	2.37	[0.91,6.13]	NS

¥ One patient with “not evaluated” as outcome was excluded from the analysis.

# = P-value ≤0.05 is statistically significant, aOR = adjusted OR, CI = confidence interval, FL = first line, LTFU = lost to follow-up, OR = odds ratio, RR-TB = rifampicin-resistant, SL = second-line, TB = tuberculosis

## Discussion

The roll out of the all-oral regimen recently recommended by the WHO in a country like Iraq, recovering from the devastating impact of prolonged conflict and disruption of the health system, is prone to face several implementation challenges. The shortage of trained healthcare personnel at the peripheral level of care hampered the early diagnosis and management of RR-TB. Only one specialized treatment centre located in the capital city provided RR-TB care, therefore making patient-centred care approach impossible and reducing access to quality care in the rural areas of the country. Also, unavailability of point-of-care DST for new drugs, the fear to adequately manage cardiotoxicity related to the use of BDQ, and the absence of clear guidelines on how to proceed in case of treatment failure on the current all-oral regimen have further delayed the decentralization of RR-TB care.

In Iraq, the use of bedaquiline-containing all-oral regimens resulted in a high treatment success and reduced lost to follow-up despite being a conflict affected area with disrupted health services. Thus, our study provides nationwide programmatic data on the implementation of WHO guidelines, as these were updated over time.

Treatment success was achieved in 63.3% of patients using the injectable-containing regimen and in 82.1% using the all-oral regimen. The bivariable analysis showed that patients on the injectable-containing regimen had more than two times odds to have an unfavorable treatment outcome, a finding not observed in the multivariate analysis. This may be due to a lack of power to detect statistical significance with our relatively small sample size.

This high treatment success using the all-oral regimen, above the current global average of 60% [1], may at least partially be explained by the patient support packages, the close technical support given by MSF, and perhaps the commitment from local partners. Few patients were lost to follow-up (LTFU) as of 2020. This is coherent with findings from other studies showing that the use of patient support strategies (i.e., adherence counselling, transport, food support and tracing mechanism) improved overall clinical outcomes for RR-TB patients [25–27]. On the other hand, incarcerated RR-TB patients received the injectable-containing regimen without adverse event monitoring nor access to regular assessment by the expert clinician, neither benefiting from a support package. This finding calls for the NTI to provide the new RR-TB package of care to this vulnerable population.

Whether a relatively low prevalence of FQr contributed to this result is unclear, as there was a high frequency of missing results for FQ DST. It was not possible to assess whether or how adverse events differed between patients treated with either the injectable-containing or the all-oral regimen as safety data was incomplete [28]. The high treatment success observed with the long all-oral regimen was similar to success reported for either the short injectable-containing or all-oral (9–11 months) regimen [29, 30].

The proportion of RR-TB patients enrolled on the all-oral regimen in 2021 increased markedly compared to 2020 (75.5% versus 53.2%). This could be explained by the clear indications established during the roll-out of this new regimen in the country. Indications included children, pregnant women, patients with co-morbidities, such as diabetes, and those with additional resistance to either fluoroquinolone or second line injectables. Moreover, clinicians gained confidence in the safety profile of BDQ and its effectiveness [17, 30].

A clinical diagnosis, without bacteriological confirmation of RR-TB, was made in 9.3% of patients. Allowing clinicians to make a clinical diagnosis important, especially in a context with poor access to DST. However, almost half had an unfavorable treatment outcome. Possibly the clinical diagnosis of RR-TB was made when patients were already in the advanced stage of the disease.

Male patients had two-fold higher odds of having an unfavorable treatment outcome compared to female patients. Other studies conducted in China and Pakistan reported a similar scenario [31, 32]. A plausible explanation for this finding specific to Iraq is the fact that men are culturally head of the household and breadwinner. This means that missing a day of work to access treatment becomes a barrier to adherence. Qualitative research to explore gender discrepancies with regards to access to care is recommended to facilitate development of targeted interventions.

In Iraq, children under 15 years were eligible for the all-oral regimen during the transition period, and they continued to receive this regimen thereafter. Nevertheless, younger age (<15 years) was significantly associated with having an unfavorable treatment outcome, as 50% of children died. This contrasts with several previous studies [33, 34]. It remains unclear, through this study, why children had worse outcomes, as we were unable to analyse possible data that could have affected their outcome such as diagnostic delay, adverse events and their management, or malnutrition. Other studies assessing underlying factors potentially affecting unfavorable outcomes in children are needed to allow better informed and targeted interventions. Meanwhile, capacity building for health care professionals dealing with paediatric RR-TB and re-enforcement of a multidisciplinary approach, linking paediatricians, TB coordinators and RR-TB clinicians might improve early diagnosis and treatment initiation.

There are several limitations to our analysis. As of 2020, following national indications, patients who had extensive disease and access to baseline pre-therapeutic work-up and regular monitoring did receive the all-oral regimen. More specifically, the all-oral regimen was offered mainly to patients who were not responding clinically and bacteriologically to the injectable regimen or those having TB resistant to fluoroquinolone or second-line injectable. This might have created selection bias. Due to the cardiotoxicity of bedaquiline and the fear of manipulating a new drug, all patients under the all-oral regimen received a closer follow-up, which might have resulted in survivorship bias. Moreover, due to the retrospective nature of this study, we could not assess causality, which would have required randomization to either the “all oral” or the “injectable-containing” regimen. As patients treated with all-oral regimens were mainly treated during more recent years, we cannot exclude that a general improvement of care during the all-oral regimen implementation contributed to the improved treatment success. Due to the small sample size, we could not obtain a precise estimate of the effect of using either an all-oral or injectable-containing regimen on treatment success. Missing data for relevant variables, such as FQ DST and AEs limited the assessment of the impact of these variables on the final treatment outcome.

This baseline study is the first of its kind in Iraq as it provides useful information to the NTI progress and performance during roll-out of RR-TB new regimens. It can, therefore, serve as a basis for additional operational research in this setting. In addition, the evidence can be used as an advocacy tool to support the transition to shorter regimens currently recommended in a context like Iraq [35, 36]. At the time of this study, enrolment and treatment initiation was done at the NTI. We believe it’s relevant to examine if the decentralization of RR-TB treatment and care strategies, successfully implemented elsewhere to increase RR-TB treatment uptake while reducing the burden associated with a long-term follow-up of patients, could also be implemented in Iraq [37–39].

## Conclusion

RR-TB management using WHO updated recommendations showed promising results in a conflict setting such as Iraq. Children and men were at risk of having an unfavorable outcome. Challenges to be addressed include decentralization of treatment services. Access to shorter regimens for all RR-TB patients may enable such decentralization. Moreover, safety data collection, access to point-of-care drug susceptibility testing and support package for all patients should improve.
